# Co-creation on Redefining Consumer Well-Being Needs Among Youth Through Self-Potential Development Model

**DOI:** 10.3389/fpsyg.2022.814757

**Published:** 2022-03-10

**Authors:** Ahmad Umair Zulkefli, Muhammad Ridhuan Tony Lim Abdullah, Mohd Nuri Al-Amin Endut

**Affiliations:** Department of Management and Humanities, Universiti Teknologi PETRONAS, Seri Iskandar, Malaysia

**Keywords:** co-creation approach, self-potential, youth, youth development, Fuzzy Delphi Method (FDM), interpretive structural modeling (ISM)

## Abstract

A co-creation values consumers’ input as its primary crust in informing businesses on current consumer needs. More importantly, it would be the next shape in future demands of consumers in business sustainability. This paper addressed this context, narrowing its scope in investigating the voices of stakeholders on what would be the essential aspects of the present and future youth qualities in achieving sustainable well-being in the present trend. The findings would be essentially helpful for the youth and the business world to understand the aspects of good youth development, which would shape the next fabric of consumerism. Self-potential development of the youth is vital in achieving excellent life quality and the youth’s well-being in Malaysia. However, the increase in challenges faced by today’s youth is inconsistent with a decline of the group’s well-being. There are various studies and interventions implemented to overcome the youth situation. However, there is still in need for a model that can guide the holistic development of youth self-potential. The purpose of this study is to build a sustainable and comprehensive model of the self-potential development of the youth, which can be integrated with all of the self-potential indicators of the youth *via* a co-creation process. This study used the Fuzzy Delphi Method (FDM) on the proposed elements in the development model and systematically analyzed them using Interpretive Structural Modeling (ISM) to create the development model. The method capitalized 10 stakeholders from various youth development backgrounds in developing the model. The model consists of 25 sub-indicators (SIs, elements) that are divided into five indicators. The model findings show that one of the most driving indicators is an entrepreneurial mindset among youth, followed by the other indicators of youth self-potential development. The model also shows that the civic-mindedness indicator is the output of youth self-potential that will surface at an end of the development. The model will guide the authorized body on the priority elements that can systematically and strategically improve youth self-potential to meet future challenges with youth aspirations.

## Introduction

Youth is a vital resource for the nation, contributing significantly to the country’s economic, social, and political advancement. Youth is the country’s future leader and successor. Without a doubt, the youth’s strength dictates the country’s strength. The youth is defined under the Youth Societies and Youth Development Act 2007 as 15–40 years (Undang-Undang Malaysia, 2007). However, in 2015, the Malaysian Youth Policy (MYP) redefined the youth as people between 15 and 30 years. Malaysia had 14.7 million youths, accounting for 46% of the country’s 31.7 million population (Department of Statistics, 2015). The data indicate that about half of Malaysia’s population is dominated by youngsters.

Youth development is critical for today’s youth, adapting to their present and future circumstances. For example, in 2019, the projected young unemployment rate in Malaysia was 11.67% (statista.com, 2020), with 22.6% of 2,759 respondents reporting that their income is inadequate to meet daily necessities (IYRES, 2018). According to the statistic by The Royal Malaysia Police (RMP) and the Ministry of Home Affairs (MOHA), the average yearly crime rate from 2013 to 2018 is shockingly high, with 87,528 cases (Institute for Youth Research Malaysia [IYRES], 2019). In addition, a survey on social behavior among 70,584 youths of 13–17-year-olds revealed that 7.3% of them already had sex, 20% were under depression, 40% were suffering from anxiety, and 10% had chronic stress. More shocking facts, 11.2% of them had an ideation of suicide, 9% had already planned on doing it, and 10.1% had already attempted (Institute for Public Health [IPH], 2018). If these issues persist, the future of the youth will be more uncertain.

In Malaysia, acknowledging the importance of the youth as an asset of the country, the Malaysian Ministry of Youth and Sports has used an index instrument, that is the Malaysian Youth Index (MYI), as a measurement tool or benchmark to monitor the development of the quality of life and well-being of the youth. The indicators used in MYI were aimed at measuring Positive Youth Development (PYD), where the youth was regarded as an assets and not a liability. Based on 3 years’ patterns from 2015 to 2017, the index score has fluctuated at a moderate level, where an aspect that needs to be improved is youth self-potential.

Even though youth policies and indexes were monitoring the youth qualities, sustainable development, which is vital for all stakeholders, is still lacking (Singh and Panackal, 2017). Conversely, despite the profound implications of these findings, most of the existing interventions of youth self-potential development seem to be conducted in isolation in terms of the impact of the youth outcome (Ciocanel et al., 2017). There is still a lack of interventions developed based on an integrated youth development framework (Cheah, 2019). Despite a tremendous impact of these programs, the isolation approach would lead to an inconsistency in the self-potential development of the youth as the continuity of the values that were tried to be instilled was not efficiently provided (Naert et al., 2017). Furthermore, these interventions did not highlight the objective of the self-potential development of the youth as a whole. Therefore, there is a need for an integrated framework to establish a continuity in the planned self-potential development programs to ensure a maximum impact on the youth target groups.

It may be apparent to the stakeholders, but the youth did not clear what they need to achieve to develop their potential continuously (Worthman, 2011; Hoaeane, 2019). It shows that a kind of predetermining guideline that the stakeholders and the youth can understand is essential to make the intervention work accurately (Bateman, 2005; Bolding et al., 2020). Thus, it is a challenge to the stakeholders to formulate policies and strategies to respond to the sustainability of youth development as it is the priority agenda of the world’s leading nations (Zu, 2020).

In addition to the current situation of the COVID-19 pandemic that happened in late 2019, the youth are most significantly affected. The youth face new challenges: unemployment, limited job, online education, economy downturn, mental health, and digital gap. The youth development model was vital as there is a need to respond to the youth disillusionment due to the pandemic. The need for a “Neo-Youth 2030” model to help the youth facing the pandemic era was emphasized by the Malaysian Youth Council (MBM) during their latest Conference on 30 June 2021 with the theme of Future-Ready Youth (Joha, 2021).

Thus, the model is vital to enhance the resilience and competency toward a brighter future, align with Concept III in MYP DBM 2015–2035, which is Futuristic, Relevance, and Up to date. MBM also founded the Youth Improvement Philosophy (FPB) as the true philosophy that navigates the stakeholders such as KBS and MBM for youth development. The term “improve” is used to develop good values and erase the terrible embedded values. It also accompanies physical youth development such as employment, entrepreneurship, personality, and internal development aspects such as morals and leadership ethics (Joha, 2021).

### Co-creation

Co-creation is a kind of management planning, or an economic strategy, in which diverse stakeholders collaborate to generate a mutually beneficial outcome. Pfitzer et al. (2013) believe that multi-stakeholder participation in the creation can understand the demand, conducive to the realization of the enterprise strategy. Since then, about the connotation of value-creating, many scholars and research specialists have attracted attention in different areas from the perspective of service innovation of science and consumer culture. The research shows that value co-creation must always focus on customers, employees, enterprises, and other stakeholders (Fan et al., 2020).

The concept of value co-creation comes from a service-dominant logic. Vargo et al. (2008) proposed the definition of value co-creation from the perspective of service science, pointing out that value co-creation is an integration of the existing resources of the service system and the resources of other service systems under certain circumstances, which is beneficial to the welfare of all parties. Customers’ participation in co-creation mainly involves the psychological motivation and personal characteristics of customers’ participation in value co-creation and related research (Roberts et al., 2014). Furthermore, more studies have shown that value co-creation affect a new design in the impact results of value co-creation. Performance, loyalty, and other aspects will have a positive impact (Wong and Lai, 2019). Different research perspectives believe that the value type of the value co-creation output is different, and some literature studies have shown that value co-creation has a “double-edged sword” effect (Chan et al., 2010; Gummerus and von Koskull, 2015; Werner, 2016).

In the context of this study, the predetermined guidelines were co-created in a focus group by a collaboration of diverse experts to generate a mutually beneficial outcome and a holistic model of youth self-potential development. This study will first suggest the proposed sub-indicators (SIs) to develop the indicators and have them validated by the experts. The indicators were initially adopted from the Self-Potential Domain of MYI. The expert focus group will then provide positive suggestions for adjusting the structure of the proposed indicators. As it turned out, there were five indicators agreed upon, namely leadership, knowledgeable, civic-mindedness, entrepreneurial, and creative and innovativeness. Then, the validated SIs will be prioritized based on experts’ views by using the Fuzzy Delphi Method (FDM). The final step will be proposing an implementation model to develop youth self-potential using an expert’s input through Interpretive Structural Modeling (ISM).

Stakeholders can plan the intervention activities more efficiently with the systematic framework guideline. The youth themselves can use the guideline to plan for their self-potential improvement, as discussed before (Forrester, 2000; Hoaeane, 2019; Bolding et al., 2020). In this study, the guideline can be created by having all of the SIs systematically sorted out with the help of the experts in the field of youth development. It can be presented in a graphical form of the implementation model that shows an operating sequence of main factors to understand the logic of their relationship. According to Edmonds et al. (2019), a model is a valuable tool in understanding their relationships that help organizations develop their decision-making processes.

### Theories of Youth Self-Potential Development

Developing youth self-potential is one of the primary purposes of PYD (Benson, 2006). Self-potential is closely related to self-development as it is used in personality psychology (Barlow, 2016). Self-potential in general refers to an individual’s strength and ability that can be unleashed to an optimum level. Meanwhile, the definition of self-potential in this study was adopted from the concept of self-potential in PYD as “the capacity of youth to change and to change in a direction that fosters both an individual’s well-being and the social good” (Benson, 2006).

Based on a previous study of PYD, self-potential can be understood as the human capacity to pursue talent (Benson et al., 2007), power yet to be (Moran, 2020), identifying the true self, life purpose and direction (Schlegel et al., 2011), passion in oneself (Arnold, 2018), and being creative in all aspects of life (Maslow, 2013). A broader perspective has been adopted by Moran (2020), who argues that self-potential is not only focusing on oneself but also should have an impact on the communities.

Developing youth self-potential can help the youth gain momentum in worthy long-term pursuits (Dai, 2010; Moran, 2020) and make clear life purposes (Dai, 2014). It also works as an intrinsic fuel for a young person’s growth in knowledge and skills (Scales et al., 2011). It will also benefit the young persons in a larger society where communities benefit through their members’ dedication to civic engagement, service, community improvement, and helping each other (Rockenbach et al., 2014). Moreover, it also enhances a young person’s network and develops both individuals and communities (Moran, 2020). It is consistent with this study’s definition where having a well-developed self-potential will help the youth improve themselves and a larger society.

**FIGURE 1:**
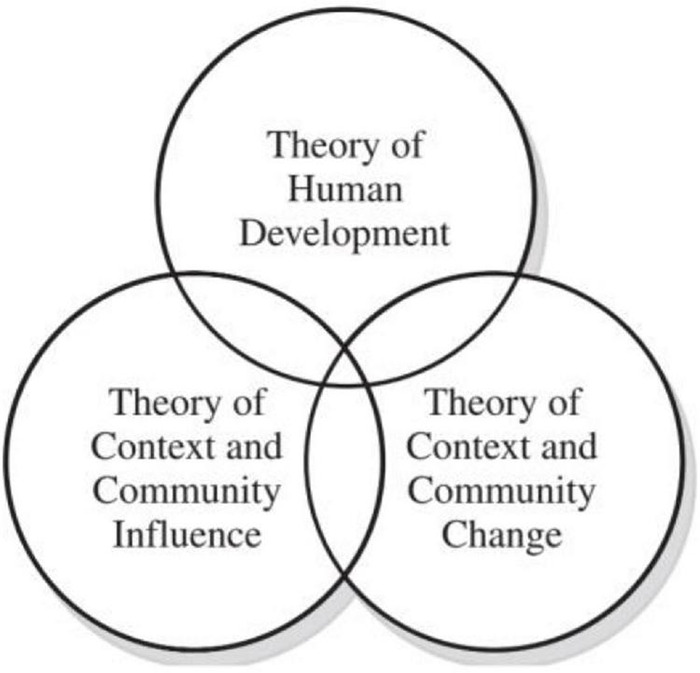
A comprehensive theory of positive youth development (PYD) (Benson, 2006).

The theory of youth self-potential development in this study is derived from the grand theory of PYD, which requires multiple theoretical orientations. In part, this is because PYD is a “bridging” field that touches multiple academic disciplines and spheres of practice. Three theoretical strands central to PYD are discussed in this section, with primary emphasis on the first. These three are human development, community organization and development and social and community changes as depicted in Figure 1 (Benson, 2006).

In the theory of human development, the central to PYD is the discipline of developmental psychology that is self-potential. The overarching goals of this theory explain the definition of self-potential: the capacity of the youth to change and to change in a direction that fosters both an individual’s well-being and the social good. Conditions such as contextual and ecological factors contribute to this change and how these factors are informed or influenced by the developing person; and the principles and mechanisms are at play in maximizing a dynamic and developmentally constructive interplay of the context and individual (Benson and Saito, 2001).

The aim of self-potential development in this study is consistent with the theory. The essential to PYD theory is a generous view of human capacity and potential. Grounded initially in the views and values of professionals and practitioners working with youth, this vision of human nature identified the possibility of an active and a constructive contribution to the development of self, a community, and the society. Such a view is often characterized in youth development circles by describing young people as resources to be nurtured vs. problems to be managed. This view is an important starting point for the PYD theory. It brings to the fore the notion that the individual—and not just the environment—is a prime actor in shaping positive developmental trajectories (Lerner and Steinberg, 2009).

A recent variant of PYD is the community youth development approach (Hughes and Curnan, 2000; Perkins et al., 2001). The approach builds on the PYD model, emphasizing engaging young people as contributors to and active shapers of their communities. As Perkins et al. (2001) argue, “community youth development shifts from a dual focus on youth being problem-free and fully prepared, to a triadic focus for youth being problem-free, fully prepared and engaged partners.”

The theory of human development, blended with the community influence and community change theory, has played its role for the youth to thrive in their lives and contribute to the society. In the context of this study, the human theory has contributed several elements to the model: knowledgeable, creative, and innovative. On the other hand, the community influence theory has contributed to leadership and civic-mindedness. Meanwhile, the community change theory has contributed to an element of entrepreneurship. All these elements, which were extracted from the self-potential theories and definition, will be presented in detail.

### Leadership

Nurturing the self-potential by understanding the true self will help the youth gain momentum in worthy long-term pursuits (Dai, 2010; Moran, 2020). Consistent with the self-potential element, knowing oneself, having a mental picture communicated, fostering trust among colleagues, and taking an effective action to realize one’s leadership potential are all necessary components of effective leadership (Hendricks et al., 2010).

Leadership is defined in this study as the ability to become a positive agent of change in the society. The youth are considered as essential assets for the well-being of a nation with the ability to bring changes, contribute, and bring positive shifts by fighting against corruption, bribes, and every social ill (Singh and Panackal, 2017). Leadership is also measured by the ability to lead others effectively. The youth should be a good, an empathic, and respected team leader who can easily lead others with action-driven quality (Williamson, 2002). Besides, the youth should also be responsible for planning and strategies to develop the youth and the entire community. As Kim et al. (2020) proposed, leadership is measured as the commitment to cooperate with others through youth collaborative communication to share diverse facts and viewpoints, including suggestions, critiques, and solutions. Leadership among the youth is also measured by channeling personal views of specific issues to the appropriate authority or parties, self-discipline, and grasping principles.

### Knowledgeable

It was also argued that the true self is a cognitive schema that contains personally meaningful knowledge (Schlegel et al., 2011). According to Benson and Scales (2009), sparks known as self-potential in their study provide an intrinsic fuel for a young person’s growth in knowledge and skills. Thus, youth self-potential can also be nurtured by having a growth in knowledge. It is measured by the youth’s desire to seek knowledge, how they demonstrate wisdom and display maturity. The youth should be empowered through education in order for them to contribute toward community development (Harrison et al., 2005). Education is needed to become knowledgeable, responsible, socially skilled, and contributable (Greenberg et al., 2003). Knowledge is essential because learning and growth are constant, never-ending, progressive, and lifelong processes. Thus, for adolescents to mature, they must continually learn, unlearn, and relearn. Indeed, what they acquired today may be irrelevant tomorrow, and they will need to unlearn previous knowledge and gain new skills required by a new environment. Finally, by the time adolescents reach adulthood and maturity, learning has become self-directed, habitual, and lifelong, making it necessary and crucial to continue learning throughout the transitional era of adolescence. It may either result in a bright and prosperous future for them or a negative and an unsuccessful future (Lee et al., 2012). In order to contribute, the youth need to have the attitude of being alert and updated with current issues.

### Civic-Mindedness

Youth civic-mindedness is a crucial factor that drives individual efforts to help others, as seen by behaviors like volunteering, aid work, and philanthropy (Ma, 2012b). Consistent with the definition of self-potential in this study, civic-mindedness will foster an individual’s well-being and the social good (Benson, 2006). Civic-mindedness is mentioned in psychological theories about self-potential, personality development, and human growth across time and sociological views about how people fit into and impact the society. According to Ma (2012a), the development of behavioral competence is strongly linked to positive personalities, with positive personalities promoting moral behavior and positive motivation triggering and maintaining good behavior.

According to psychologists such as Erikson (1993), understanding one’s roles and connections to a larger society is crucial for a healthy psychosocial development and a facet of successful identity building in adolescence. According to the humanist psychology approach, civic-mindedness is a manifestation of self-actualization, an individual’s evolving capacity to identify with, respect, and support the welfare of other human beings, according to the humanist psychology approach (Maslow, 1972). According to sociologists, civic-mindedness is an essential component of “social capital” because it contributes to the culture of trust and reciprocity that underpins social relationships and networks and facilitates a collective action and civic involvement (Winter, 2000).

Civic-mindedness among the youth is crucial to being the part of a good citizen. Civic-mindedness has been characterized as an individual’s readiness to actively assume the role of a citizen (Bowes et al., 1996). This concept emphasizes both a broad and personal concern for others’ well-being. In this study, this is portrayed by having a positive interaction such as *adab*, prudent, manners, courtesy, and politeness with all society members as a personal practice. Besides, civic-mindedness is also portrayed by respect toward others in the society, aiming to ensure a peaceful and conducive environment in a harmonious society (Smart et al., 2000).

Furthermore, the youth must accept and tolerate the differences in religion, culture, and the society (Munardji et al., 2020) by becoming open-minded and ready to accept variances. Moreover, the youth should have a well-developed common sense toward all social levels and environments (Smart et al., 2000). Common sense is a fundamental capacity for seeing, comprehending, and judging the things that practically all individuals share. Another asset for the youth is an appreciation toward individuals, groups, and a collective culture. By having civic-mindedness, the youth will demonstrate ethics, especially in this unbounded digital world they live in, where anybody can freely express their thoughts and feelings.

### Entrepreneurial Skills

Entrepreneurial skills should not only be confined to owning a profiting business or company as the act should also include the attitude, spirit, competence, and mentality typically seen in an entrepreneurship (Nguyen et al., 2019; Kim et al., 2020). It is defined by Small and Memmo (2004), who argued that an entrepreneurial indication is characterized by the resilience and the capacity to manage risks to achieve the success in life *via* meticulous preparation and hard work. It is in line with the definition of self-potential in this study, which is “the capacity of youth to change and to change in a direction that fosters both an individual’s well-being and the social good” (Benson, 2006).

Another essential component that the youth should empower themselves is having an entrepreneurial mindset and character (Olugbola, 2017). In light of today’s economic downturn, which has resulted in a high proportion of youth unemployment, entrepreneurship has emerged as a widely recognized tool for alleviating poverty worldwide (Saptono et al., 2020). In contrast to Kim et al. (2020), entrepreneurship has a relatively broad definition encompassing entrepreneurial mindsets, skillsets, and aspirations.

Many academics have described entrepreneurial orientation in unique ways; the most frequently accepted approach is Miller’s three indicators: innovativeness, risk-taking, and proactiveness (Kim et al., 2020). The youth should be interested in becoming a successful entrepreneur by having the mindset of self-employed who earns the income directly from his own business. Besides, the youth should have the ability in exploring entrepreneurial opportunities, not only limited to the conventional business sectors but also the e-economy. Entrepreneurial skill is also measured by the willingness to diversify income-earning and marketable skills with more than one core competence and the ability of the youth to generate new entrepreneurial values in the society.

### Creative and Innovativeness

Consistent with the definition of self-potential of being creative in all aspects of life (Maslow, 2013), creativity and innovativeness are the elements that need to be empowered by youth. Numerous studies have shown that the youth with critical and creative thinking skills performed better in schools and colleges (Phan, 2008; Lun et al., 2010), had better health (Irving et al., 1998; Rindner, 2004), and had a better cognitive development (Zhang, 2002). They also have a better psychosocial development (Zhang, 2010) and identity development (Zhang, 2008) and were less likely to engage in unhealthy or problem behavior (Kater et al., 2000; Scull et al., 2010). As a result, critical and creative thinking are seen as generic, transferable life skills for teenagers (Feldhusen et al., 1970; Speedie et al., 1971; Segal et al., 1985; Edward, 1991), who face various developmental stresses and problems. Critical thinking entails reasoning and inferences, while creative thinking entails widening one’s horizons, weighing numerous ideas and options, and coming up with new and practical ideas (Sun and Hui, 2012).

Both indicators are measured by generating new ideas to solve problems, where the youth can think of new and practical ideas to solve problems (Jedaman et al., 2018). In addition, the youth should have empowered by the ability to treat an issue from different perspectives while making a decision. They should have a dynamic thought process based on reference dependence and various perspectives. Moreover, the ability to think critically and analytically is also required among youth, where they could have the agility in thinking and be able to create and produce ideas. The youth are also expected to improve or enhance the intervention of an issue or a process.

All five indicators discussed above are the core indicators of youth self-potential and essential to be developed. Numerous studies and interventions were implemented to develop youth self-potential. However, there is still in need for a model that can guide a holistic development of youth self-potential, integrating and relating all of its indicators.

This study aims to propose and validate the elements of youth self-potential *via* FDM. It will then suggest a sustainable and holistic self-potential development model for the youth that integrates with all the indicators of youth self-potential prioritized by experts’ opinions in FDM systematically developed using ISM. The methods are explained in Section “Materials and Methods.”

## Materials and Methods

This study aims to develop a self-potential Development Model for the youth through a co-creation approach. The model was constructed using the aggregated opinions of different stakeholders from various backgrounds. To manage the decision made by the stakeholders to develop the model, the FDM and ISM were used as the primary methodologies of this study. In the context of this study, FDM was employed in youth development to determine the level of agreement among experts about the proposed SIs for the Youth Self-Potential Indicators and ISM was employed to determine the prominent SIs of the agreed SIs.

Fuzzy Delphi Method is a method introduced in 1985 by Murray, Pipino, and Gigch and evaluated in 1988 by Kaufman and Gupta (Rosman et al., 2015). This approach combines the conventional Delphi method with fuzzy theories to enhance the Delphi method’s ambiguities (Ishikawa et al., 1993). FDM is not a new technique as this technique is already emerged in the research area since the 1980s. However, FDM is an improved method of the traditional Delphi method by integrating Fuzzy Theory to overcome uncertainties, vagueness, ambiguities, fuzziness, and imprecision (Mohd Ridhuan et al., 2015).

Interpretive Structural Modeling is an analytical technique that enables people or groups to construct a framework for all existing interactions between the many components of a complex system (Warfield, 1974). The fundamental purpose of this approach is to use specialists’ knowledge and expertise to analyze complex system challenges and then create a multi-level structural model (Bolaños et al., 2005; Singh and Kant, 2008).

The Fuzzy Delphi Method and ISM have been used multiple times in youth research. For example, FDM has been used in research to establish a model for developing the entrepreneurial abilities of urban adolescents in China *via* community-based leadership training (Sai et al., 2019). Other researchers have studied the priority skills required for the electrical engineering student’s marketability in Malaysia by using ISM (Hasan et al., 2017). Singh and Panackal (2017) have employed ISM to study the relationship between the youth and sustainable indicators through the development model. Another research studies the relationships between sustainability and ecopreneurship in the youth by using ISM (Panackal et al., 2016).

Hence, this study employed FDM and ISM because of their advantages in using experts’ opinions and its significance for collecting the experts’ opinions to make decisions on a complicated problem. As a method for evaluating the study’s outcomes, the implication of the FDM serves as an example for future researchers to design and develop as guided. The advantages of ISM modeling, considering its holistic perspectives, as conducted in previous youth and social studies, will be an excellent potential in this study. It will provide a systematic improvement approach to developing the youth while considering all of the indicators and SIs involved in youth self-potential development.

The participants of this study are comprised of 10 experts’ panels. The panels were invited to participate in a virtual focus group held on 17 October 2020, using the Zoom application. Based on Adler and Ziglio (1996), 10–15 experts are an optimal number in a Delphi study. In this study, initially, 15 experts were invited. The invitation was issued a month before the workshop. From the 15 invitations, only 10 experts agreed to involve in the FDM workshop subject to suitability and accessibility.

The study was conducted in seven steps as follows:

*Step 1*: Experts review the proposed indicators and SIs through a focus group discussion.

The first instrument was the proposed indicators generated from the literature review. The list acted as a reference for experts in determining the critical indicators to include in the model of youth self-potential development. Before the discussion starts, the expert will be briefed about the definition of each indicator. This session is vital so that the experts can reach the same understanding before evaluating the indicators. The focus group of experts could provide positive suggestions for adjusting the structure of the proposed indicators. It would be decided if it should be included in the model, regrouped, or omitted entirely. Experts were invited to submit any additional indications they deemed appropriate for inclusion in the instrument. The experts were provided with the final list. The experts expressed their degree of preference for the items by assigning a Likert scale value between 1 and 7, where 1 represents “most least important” and 7 represents “very most important” for each SI in the FDM.

*Step 2*: Identifying elements for the model *via* FDM.

**FIGURE 2:**
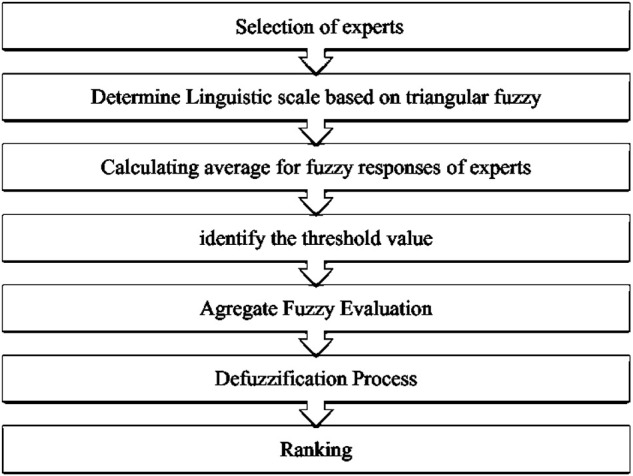
A flowchart of a Fuzzy Delphi Method (FDM) procedure.

This stage aimed to suggest and define Youth Self-Potential SIs for the development model. A flowchart of the FDM is shown in Figure 2.

The experts selected for this research consisted of policymakers, government bodies, research and education institute, non-governmental organizations (NGOs), youth, and academia as stated in Table 1.

**TABLE 1:** Expert selection for this study.

Stakeholders	Description	No of experts
Policymaker	KBS	1
Research and Education Institute	IYRES	2
NGO	Majlis Belia Malaysia (MBM)	2
	MyFundAction	1
Academia	Researcher on Youth Development	1
	Malaysian Youth Index Developer	2
Youth representative	Youth personal	1

**FIGURE 3:**
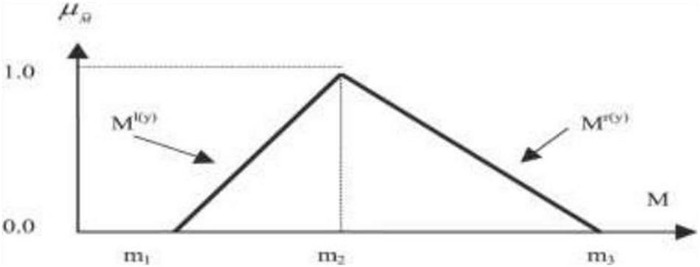
Triangular fuzzy number.

A linguistic scale is developed to contextualize respondents’ responses to alleviate the ambiguities inherent in expert judgments. The linguistic scale is similar to the Likert scale, except it includes an extra set of fuzzy numbers based on the triangle fuzzy number (as shown in Figure 3). Three fuzzy values were assigned to each answer to account for the fuzziness of the experts’ viewpoints. As seen in the accompanying picture, the three values included the three degrees of fuzzy value, namely the lowest value (m1), the most reasonable value (m2), and the highest value (m3) (m3).

The linguistic scale, in other words, is employed to transform the language variable into fuzzy numbers. The agreement scale should have odd numbers (three-, five-, or seven-point linguistic scale). In general, the larger the size, the more precise the response analysis. A seven-point linguistic scale will be used in this investigation.

There are three criteria to determine the acceptance of an indicator. However, the determination of the acceptance is executed for the three criteria simultaneously in a row.

The threshold value, “*d,”* is essential to determine the consensus level among the experts. According to Cheng and Lin (2002), if the threshold value is less than or equal to 0.2, all experts are considered to achieve a consensus. The threshold values that are “**bold”** marked in the sample calculation of the above table indicate the outliers, where the individual user’s opinions do not agree with the other expert’s view.

The experts’ agreement of an indicator should be more than 75%. However, what is more important to be considered is the overall consensus for all items. The overall group consensus should be more than 75% (Cheng and Lin, 2002); otherwise, the second round of fuzzy Delphi needs to be conducted.

The defuzzification value ‘A’, determines the ranking of the indicators among the other indicators. A low ranking of an indicator indicates low acceptance of experts’ consensus.

*Step 3*: Determine the contextual phrase and relation phrase.

This stage establishes the contextual connection and relation phrase for how the SIs (components) should be related. The contextual connection establishes the objective (goal) and any boundary conditions or limits along the route. In other words, the context sheds a light on how the SIs should be linked throughout the construction of ISM.

Before the model was obtained, a reachability matrix was constructed. From the matrix, the software can extract a multi-level digraph. The digraph is then substituted with the elements used for the study. Partitioning the reachability matrix is to classify the SIs into different levels. The partitioning is essential in the interpretation of the model at the end of this study. To obtain the reachability matrix, a structural self-interaction matrix (SSIM) was developed based on a pairwise comparison of SIs. The pairwise relationship was based on the two factors, which were i and j. Four symbols were used to denote the direction of a relationship between the two factors (i and j) (Attri et al., 2013). The description for each symbol is as follows:

(a) V for the relation from the factor i to the factor j (i.e., the factor i will influence the factor j).
(b) A for the relation from the factor j to the factor i (i.e., the factor i will be influenced by the factor j).
(c) X for bidirectional relations (i.e., the factors i and j will influence each other).
(d) O for no relation between the factors (i.e., the factors i and j are not related to each other).

From the SSIM, the initial reachability matrix was obtained by substituting the symbols (V, A, X, and O) with 1 or 0. The processes of substitution were as follows:

(a) If the (i, j) entry for SSIM is V, then the (i, j) entry in a reachability matrix would become (i, j) = 1 and (j, i) = 0.
(b) If the (i, j) entry for SSIM is A, then (i, j) entry in a reachability matrix would become (i, j) = 0 and (j, i) = 1.
(c) If the (i, j) entry for SSIM is X, then (i, j) entry in a reachability matrix would become (i, j) = 1 and (j, i) = 1.
(d) If the (i, j) entry for SSIM is O, then (i, j) entry in a reachability matrix would become (i, j) = 0 and (j, i) = 0.

After all, relationships had been substituted, and a reachability matrix was obtained. From this final reachability matrix, factors were classified into an antecedent set and a reachability set. The antecedent set comprises SIs and other factors that may help achieve it. In contrast, the reachability set comprises factors and other factors that it may help achieve (Attri et al., 2013). The intersection of both sets was derived for all SIs. The SIs for which the reachability and the intersection are the same present the top level among the other factors in the ISM process. Top-level factors are also the most influential factors which drives other factors at lower levels. When the top-level factor is determined, the factor was put aside from consideration. The process was repeated for other factors. When all factor levels have been determined, the digraph and model can begin to be built.

*Step 4*: Develop SSIM using the ISM software.

At this stage, a SSIM of the SIs (elements) is produced to illustrate their relationship. This technique made use of the ISM software. The program would show pairs of items to enable experts to vote on the relationship before displaying the next pair of components. This procedure is done until all components have been linked to form a connection.

*Step 5*: Generate ISM.

The software carries out this stage after a successful pairing of elements. The model is derived by the program using a pairwise comparison and transitive logic concepts. When any three items (A, B, and C) have a specific relationship, a transitive logic asserts that:

• A has the relation to B, (written A → B),
• And, B has the relation to C, (written B → C),
• Then, A has the relation to C, (written A → C or A → B → C).

*Step 6*: Conducting a conceptual review of the model and making appropriate adjustments.

*Step 7*: Final model.

## Results

The results will be presented by stating the findings from each step.

### Findings From Step 1

Based on the experts’ discussion, five (5) indicators and twenty-five (25) SIs are proposed in the self-potential development model for the youth. Initially, the indicators were adopted from the Self-Potential Domain in MYI. Throughout a discussion among the experts, it was agreed that the indicators and SIs of youth self-potential should not be limited only by the domains in the MYI. The finalized indicators and SIs proposed by the experts are presented in Table 2.

**TABLE 2:** Proposed indicators and sub-indicators (SIs) for youth self-potential.

Indicator	No	Sub-indicator
Leadership	1	Ability to become a positive agent of change to society.
	2	Ability to lead others effectively.
	3	Ability to plan and strategies for youth and community development.
	4	Commitment to cooperate with others.
	5	The ability to channel personal views of specific issues to appropriate authority or parties.
	6	Having self-discipline and being consistent in grasping principals.
Knowledgeable	7	Desire in seeking knowledge.
	8	Demonstrate wisdom.
	9	Display maturity.
	10	The attitude of being up to date with current issues.
Civic-mindedness	11	Having a positive interaction such as *adab*, prudent, manners, courtesy, and politeness with all society members as a personal practice.
	12	Having respect toward others in the society.
	13	Acceptance and tolerance of differences in religion, cultural and social
	14	Have a well-developed common sense toward all social levels and environment.
	15	Appreciation toward individuals, groups, and collective culture.
	16	Demonstrate ethics.
Entrepreneurial	17	Interested in becoming a successful entrepreneur.
	18	Ability in exploring entrepreneurial opportunities.
	19	Resilient and the ability to manage risks to succeed in life.
	20	Willingness to diversify income-earning and marketable skills with more than one core competence.
	21	Ability to generate new entrepreneurial values in society.
Creative and innovativeness	22	The ability to generate new ideas to solve problems.
	23	The ability to treat an issue from different perspectives in making a decision.
	24	The ability to think critically and analytically.
	25	Ability to improve or enhance an intervention to an issue or process.

### Findings From Step 2

Based on the result of the FDM, the experts agreed with all the 25 proposed SIs with a 93% consensus. The 93% consensus is due to a high understanding and agreement among the experts after going through Step 1. They proposed the SIs together and agreed on it.

The indicators were arranged based on the highest Defuzzy number and the most prioritized indicators that portrayed youth self-potential. The five top SIs are desired in seeking knowledge (9.75), having a positive interaction with all society members (9.55), followed by the ability to think critically and analytically (9.55), demonstrate ethics (9.45), and having resilience and able to manage a risk to succeed in life (9.45).

Table 3 presents the expert collective views of the youth self-potential SIs, which should be included in the development model using the FDM.

**TABLE 3:** Summary of the results of FDM.

Ranking	Number of elements	Content element	Defuzzy	*d*
1	7	Desire in seeking for knowledge.	9.75	0.000
2	11	Having a positive interaction such as *adab*, prudent, manners, courtesy, and politeness with all society members as a personal practice.	9.55	0.041
3	24	The ability to think critically and analytically.	9.55	0.041
4	16	Demonstrate ethics.	9.45	0.054
5	19	Resilient and the ability to manage risks to succeed in life.	9.45	0.054
6	9	Display maturity.	9.35	0.062
7	4	Commitment to cooperate with others.	9.35	0.062
8	13	Acceptance and tolerance of differences in religion, cultural and social.	9.275	0.077
9	14	Have a well-developed common sense toward all social levels and environment.	9.275	0.077
10	2	Ability to lead others effectively.	9.25	0.065
11	6	Having self-discipline and being consistent in grasping principals.	9.25	0.065
12	8	Demonstrate wisdom.	9.25	0.065
13	15	Appreciation toward individuals, groups, and collective culture.	9.15	0.062
14	22	The ability to generate new ideas to solve problems.	9.15	0.062
15	20	Willingness to diversify income-earning and marketable skills with more than one core competence.	9.075	0.082
16	18	Ability in exploring entrepreneurial opportunities.	9.05	0.054
17	12	Having respect toward others in the society.	8.975	0.076
18	25	Ability to improve or enhance an intervention to an issue or process.	8.975	0.076
19	1	Ability to become a positive agent of change to society.	8.875	0.066
20	23	The ability to treat an issue from different perspectives in making a decision.	8.875	0.066
21	3	Ability to plan and strategies for youth and community development.	8.775	0.051
22	21	Ability to generate new entrepreneurial values in society.	8.7	0.080
23	10	The attitude of being up to date with current issues.	8.6	0.069
24	17	Interested in becoming a successful entrepreneur.	8.4	0.099
25	5	The ability to channel personal views of certain issues to the appropriate authority or parties.	7.975	0.099

According to Table 3, the FDM session demonstrates that the experts unanimously agreed on all the suggested Sis (components) for the design of a structural model.

### Finding From Step 3

Based on the agreed upon Sis for youth self-potential, the experts determined that the phrase “In the effort to develop youth self-potential, the SI will significantly assist in developing…” is critical to guide the reader through the SSIM process, while the phrase “will significantly assist in developing” serves as the relational phrase connecting the elements of this model.

A structural self-interaction matrix developed from contextual relationships were converted into binary matrices called initial reachability matrices, by replacing V, A, X, and O by a combination of 1s and 0s in accordance with the VAXO rules. If the entry (i, j) in SSIM = “V,” enter the element (i, j) as “1” and (j, i) as “0” in the initial reachability matrix.

If entry (i, j) in SSIM = “A,” enter element (i, j) as “0” and (j, i) as “1” in initial reachability matrix If entry (i, j) in SSIM = “X,” enter element (i, j) as “1” and (j, i) as “1” in initial reachability matrix If entry (i, j) in SSIM = “O,” enter element (i, j) as “0” and (j, i) as “0” in initial reachability matrix.

The reachability matrix, as shown in Table 4, defines each SIs driving power and dependence power. Horizontally, the total numbers on the right-hand side of the table indicate the driving power for each SI. It is the total number of all SI that the SI may help to achieve, including it. Vertically, the dependence power of SIs is the total number of SIs (including itself), which may help achieve it. For example, for SI 24—interested in becoming a successful entrepreneur, the driving power is 22, indicating that this SI must be conducted before the other SIs except SIs 4, 7, and 25 are not related to it. The dependence power of SI 24 is only “1,” which only depends on itself.

**TABLE 4:** Final reachability matrix.

SI	1	2	3	4	5	6	7	8	9	10	11	12	13	14	15	16	17	18	19	20	21	22	23	24	25	DP
1	1	1	1	0	1	1	0	1	1	1	1	1	1	1	0	1	1	1	1	1	1	0	1	0	0	19
2	1	1	1	0	1	1	0	1	1	1	1	1	1	1	0	1	1	1	1	1	1	0	1	0	0	19
3	1	1	1	0	1	1	0	1	1	1	1	1	1	1	0	1	1	1	1	1	1	0	1	0	0	19
4	0	0	0	1	1	0	1	1	1	0	0	0	0	0	0	0	1	0	0	0	0	0	0	0	1	7
5	0	0	0	0	1	0	0	0	1	0	0	0	0	0	0	0	0	0	0	0	0	0	0	0	0	2
6	1	1	1	0	1	1	0	1	1	1	1	1	1	1	0	1	1	1	1	1	1	0	1	0	0	19
7	0	0	0	1	1	0	1	1	1	0	0	0	0	0	0	0	1	0	0	0	0	0	0	0	1	7
8	0	0	0	0	0	0	0	1	0	0	0	0	0	0	0	0	1	0	0	0	0	0	0	0	0	2
9	0	0	0	0	1	0	0	0	1	0	0	0	0	0	0	0	0	0	0	0	0	0	0	0	0	2
10	1	1	1	0	1	1	0	1	1	1	1	1	1	1	0	1	1	1	1	1	1	0	1	0	0	19
11	1	1	1	0	1	1	0	1	1	1	1	1	1	1	0	1	1	1	1	1	1	0	1	0	0	19
12	0	0	0	0	1	0	0	1	1	0	0	1	0	0	0	0	1	0	0	1	1	0	0	0	0	7
13	1	1	1	0	1	1	0	1	1	1	1	1	1	1	0	1	1	1	1	1	1	0	1	0	0	19
14	1	1	1	0	1	1	0	1	1	1	1	1	1	1	0	1	1	1	1	1	1	0	1	0	0	19
15	1	1	1	0	1	1	0	1	1	1	1	1	1	1	1	1	1	1	1	1	1	1	1	0	0	21
16	1	1	1	0	1	1	0	1	1	1	1	1	1	1	0	1	1	1	1	1	1	0	1	0	0	19
17	0	0	0	0	0	0	0	1	0	0	0	0	0	0	0	0	1	0	0	0	0	0	0	0	0	2
18	1	1	1	0	1	1	0	1	1	1	1	1	1	1	0	1	1	1	1	1	1	0	1	0	0	19
19	1	1	1	0	1	1	0	1	1	1	1	1	1	1	0	1	1	1	1	1	1	0	1	0	0	19
20	0	0	0	0	1	0	0	1	1	0	0	0	0	0	0	0	1	0	0	1	1	0	0	0	0	6
21	0	0	0	0	1	0	0	1	1	0	0	0	0	0	0	0	1	0	0	0	1	0	0	0	0	5
22	1	1	1	0	1	1	0	1	1	1	1	1	1	1	1	1	1	1	1	1	1	1	1	0	0	21
23	1	1	1	0	1	1	0	1	1	1	1	1	1	1	0	1	1	1	1	1	1	0	1	0	0	19
24	1	1	1	0	1	1	0	1	1	1	1	1	1	1	1	1	1	1	1	1	1	1	1	1	0	22
25	0	0	0	0	0	0	0	0	0	0	0	0	0	0	0	0	0	0	0	0	0	0	0	0	1	1
DEP	15	15	15	2	22	15	2	22	22	15	15	16	15	15	3	15	22	15	15	17	18	3	15	1	3	

*SIs, sub-indicators; DP, driving power; DEP, dependence power.*

### Findings From Steps 4–7

**FIGURE 4:**
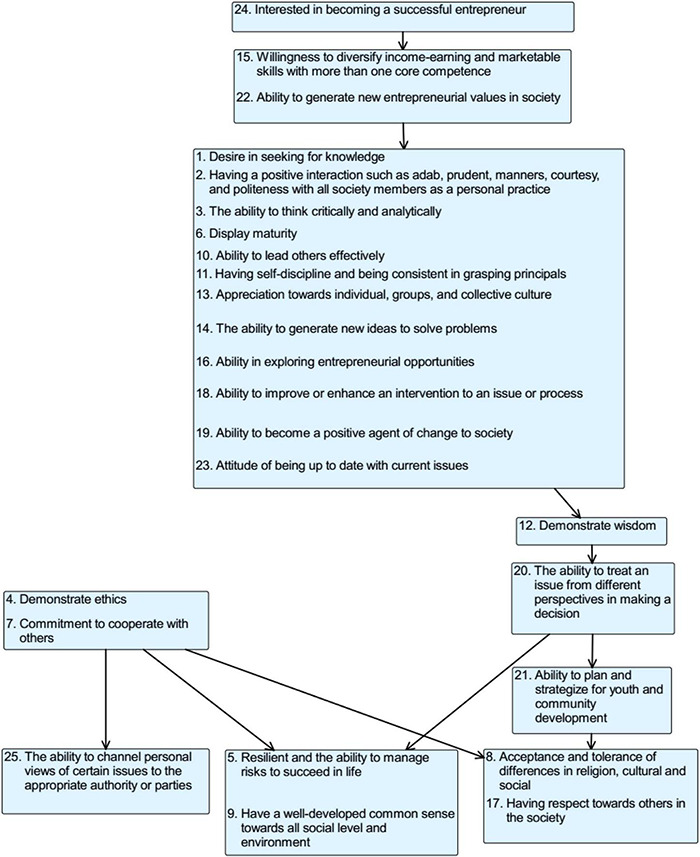
Interpretive Structural Modeling (ISM) based on youth self-potential SIs.

These phases attempt to create a model by eliciting expert judgments about the components’ connections using a pairwise procedure and the ISM software, as explained before in the methodology section. Following the generation of this model, professionals examined it, and the final model is shown in Figure 4.

The model is structural in character and is interpretively produced by the constructed experts through a network of linkages among SIs, also defined as model components. The association between the SIs was established using the contextual phrase, which was identified in Step 3 of the research. The SIs, contextual phrases, and associated phrases were found using youth self-potential indicators.

Briefly, the model can be divided into four parts of the implementation of SI development:

(a) the ignitor SIs;
(b) the resulting or the output SIs;
(c) the middle process SIs; and
(d) the supporting SIs.

The arrows indicate the movement from one SI asset to another SI asset of sequence development connected to producing an overall sequence SI structure for youth self-potential development. For instance, SI 24 must be established before SIs 15 and 22. SIs 15 and 22, SIs 4 and 7, SIs 5 and 9, and SIs 8 and 17 all share a single box, implying that the SIs may be created in any order or simultaneously as the SIs complement one another.

## Discussion

As previously stated, the model can be divided into four parts. The first part on the top is (a) the ignitor SIs. These SIs developments are the most preliminary, which need to be developed before another SIs as the other SIs are depending on them. Surprisingly, the result of this study shows that to develop self-potential among the youth in Malaysia, the Indicator Entrepreneurial is a fundamental indicator that needs to be focused on. Most experts initially believed that the Knowledgeable Indicator would be the most important; however, the model’s findings show otherwise. The Indicator Entrepreneurial might not be the most critical indicator among the five indicators. However, it will be a primary indicator that helps to develop the other indicators of youth self-potential.

Based on Figure 4, SI 24 (interested in becoming a successful entrepreneur) is positioned at the highest in the whole model to develop Malaysian youth. It then will significantly develop the next SI, SIs 15 (willingness to diversify income-earning or marketable skills with more than one core competence) and 22 (ability to generate new entrepreneurial values in the society). Considering the one-dimensional interpretation of an entrepreneur as someone who invests capital to grow a business is a common misconception among the youth (Hunter, 2012). However, being a successful entrepreneur should not only be confined to owning a profiting business or company as the act should also include the attitude, spirit, competence, and mentality typically seen in entrepreneurship (Nguyen et al., 2019; Kim et al., 2020). The youth can be entrepreneurs in different settings, including school, university, home, NGO, and society. Making profits is not the main priority, but it is about how the youth develop their marketable skills in these settings and make decisions under challenging circumstances while maintaining respect for others. It is similar to a successful entrepreneur who is sensitive to profitable opportunities and is courageous to use innovative and efficient methods to improve the profitability of their products. The quality is there in the youth who are equipped with entrepreneurial qualities and characteristics that can have the advantages of adapting to changes, staying current and relevant, and focusing on creating positive impacts on the society (Jiao, 2011; Eisenhardt and Bingham, 2017; Doh et al., 2019).

Another important finding was the (b) output phase. Interestingly, of all the 25 SIs in this study, the SI from civic-mindedness indicator determined as the final SI needs to be developed. It explains why the recent youth is usually not culturally competent and has low common sense toward others (Vargas and Erba, 2017; Grealy, 2018). The model explains that it is necessary to go through all previous SIs developments to develop these qualities. The SIs from civic-mindedness in the output phase are SI 9 (have a well-developed common sense toward all social levels and environments), SI 8 (acceptance and tolerance of differences), and SI 17 (having respect toward others in the society).

Another finding is the 15 SIs in the model’s center that falls in the (c) middle phase. Over here, there is at least one-third of each indicator. The middle phase seems to be taking the most prolonged period to be developed as this phase has 15 SIs and multiple angles to be tackled. Nevertheless, according to the model, it will be manageable if it is developed accordingly, starting with an entrepreneurial indicator in the Ignitor Phase and the SI 1 (desire in seeking knowledge) from a knowledgeable indicator. It is widely known that knowledge is power, but the factors that motivate and derive the desire in seeking it is yet to be discovered. The findings of this study are consistent with those of Bux and Van Vuuren (2016) and Saptono et al. (2020), who stated that having an entrepreneurial mindset will lead someone to explore more options to succeed. Following the present results, previous studies have demonstrated that one will be more creative and think critically to generate new ideas by having this mindset (Farhangmehr et al., 2016; Eggers et al., 2017; Mcdonald, 2017; Julien-Chinn and Lietz, 2019). It explains why SI 3 (the ability to think critically and analytically) is also suggested to be developed in this phase. Likewise, having an entrepreneurial mindset will lead to leadership quality because becoming a good leader is unavoidable to succeed in a team effort (Bressler and Sohmen, 2017; Olugbola, 2017). It indirectly explains the presence of SI 10 (ability to lead others effectively).

Contrary to expectations, this study reveals 3 SIs (i.e., the previous SIs) that do not induce in the model, which is the (d) supporting SIs. Initially, all of the 25 SIs were discussed and proposed as the element in youth self-potential. However, it turns out differently when the model shows SI 4 (demonstrate ethics) and SI 7 (commitment to cooperate with others) as it should be developed separately. It will assist in developing the output phase. This finding corroborates the ideas of Asakawa et al. (2017) and Ahmad Yusoff (2018), who suggested that consistently cooperating with others will lead to acceptance and tolerance within societies from different backgrounds.

In short, the constructed self-potential development model has been logically, hierarchically, and systematically established, and it has defined the interrelations between indicators and SIs. The model suggested that entrepreneurial mindsets should initiate the process to develop youth’s self-potential. The co-created model is a meaningful finding to improve the current youth policy framework and suggest additional perspectives to the Self-Potential Domain of MYI for future improvements. Referring to the role of the SIs in the respective clusters, the experts need to pay attention to all 25 SIs as they individually and connectedly influence the development of youth self-potential. In terms of the youth self-potential development, the classified SIs as discussed above were based on experts’ collective decisions regarding the indicators of youth self-potential as mentioned in the section findings. The model could also guide how the SIs individually and in connection help the youth development to achieve the indicator outcomes. However, the SIs are not exclusively implemented to serve a particular indicator outcome. A SI or a set of SIs could help fulfill multiple indicators during the youth self-potential development. By having the holistic and predetermine guidelines in the model, the issue of isolated and discontinuity intervention will be solved.

## Conclusion

The objective of this study was to identify SIs of youth self-potential and the usage of ISM in preparing its implementation of the development model using a co-creation approach. This study has shown that the Indicator Entrepreneurial will ignitor the development to develop Malaysian youth self-potential. It is a fundamental indicator that must be focused on and developed prior. The second significant finding was about the SIs from the civic-mindedness indicator, which was determined as the development output and would be posited in the final SIs that should be developed. The methodology described in this research may be utilized to build targeted, realistic, and successful solutions for the youth self-potential development.

The contribution of this model is to provide the input for the Malaysian youth authority bodies to gazette a FPB to answer the challenges faced by the youth in the pandemic era (Joha, 2021). The pandemic has an extreme impact on unemployment among youth, thus needing youth to instill an entrepreneurial mindset and polish their competence in entrepreneurship that will help the youth in future.

The outcomes of this study were an integrated and holistic framework to develop youth, where the prioritized SIs are shown in the model. According to the experts, the practical implications of this study are that being interested in becoming a successful entrepreneur is fundamental to the cultivation of self-potential among youth. Throughout this study, the experts agree that this finding is an important wake-up call to instilling entrepreneurial qualities among the youth by the only classroom experience. The youth need a practical and realistic platform to experience the world of entrepreneurship. They need a learning environment that poses risks and challenges their current level of understanding. They also need a forgiving culture that allows them to make mistakes and rebound from them. In this information age with many technological advances, the youth must also be empowered to connect with people of different countries, cultures, and backgrounds to facilitate exchanges of ideas and understanding of universal values. Youth development should be designed so that self-dependence is cultivated to become self-independence and interdependence. Emphasis on acquiring knowledge should transit to the ability to make connections with others and, lastly, desire to contribute to a greater good. The future youth development project should create and maintain such a culture and an environment in different settings at different age levels. Thus, the model guides the stakeholders to take prior action on the ignitor SIs to achieve the output indicators with the help of supporting and intermediary SIs. Thus, the issue of isolated, inconsistency and discontinuity improvement will be addressed as this solution.

On the other hand, this model would make a significant contribution to youth employment values through a co-creation. Co-creation platforms have mostly been utilized to improve client interaction, but they also have the potential to improve organizational changes. Co-creative platforms can help with a wide range of change goals, including technology implementation, post-merger integration, restructuring, and culture transformation (Payne et al., 2008). Organizations frequently fail to engage or consult with young people. It is possible that organizations do not recognize the value that young people can bring, or that they do not know how to engage them in a way that is both meaningful and useful. Employers would benefit from the proposed model because it would help them understand what skills and qualities the next generation of young people will need to meet future and current societal needs for goods and consumption. People used to value hunting for work and following an employer’s rules and regulations make a profit. However, as the world evolves, everyone is looking for a better way to make money and have more independence. Those who were dissatisfied with the organization’s regulations were more likely to resign than to simply follow the rules. If there is no appropriate strategy in place to keep workers, the company may go out of business. The manner in which young people interact, respond, and engage is changing at a rapid pace as well. Thus, this model would assist companies in a better understanding of the needs of young workers.

The theoretical implication of this study is the development of a conceptual framework for identifying critical indicators for the self-potential development model for the youth. The first step is to examine the concepts of youth self-potential and the theory of PYD. Three theoretical strands central to PYD are discussed in this study, with primary emphasis on the first. These three are human development, community organization and development, and social and community changes (Benson et al., 2007).

Like any study, the present study has its limitations. The study has developed a youth self-potential framework based on literature the review and validation by the experts’ decision. The research limits itself to a deduction and an understanding of the concept of youth self-potential and its core elements; however, it does not probe the nitty-gritty of the elements at a microscopic level. The study only talks about the core relations between the youth and their self-potential. However, methodically this is not a sound approach and may attract criticism as it needs to be further investigated using the survey data. The limitations of this present study can further be extended in the future.

## Data Availability Statement

The original contributions presented in the study are included in the article/supplementary material, further inquiries can be directed to the corresponding author/s.

## Author Contributions

All authors listed have made a substantial, direct, and intellectual contribution to the work, and approved it for publication.

## Conflict of Interest

The authors declare that the research was conducted in the absence of any commercial or financial relationships that could be construed as a potential conflict of interest.

## Publisher’s Note

All claims expressed in this article are solely those of the authors and do not necessarily represent those of their affiliated organizations, or those of the publisher, the editors and the reviewers. Any product that may be evaluated in this article, or claim that may be made by its manufacturer, is not guaranteed or endorsed by the publisher.
